# Relationships of brain cholesterol and cholesterol biosynthetic enzymes to Alzheimer’s pathology and dementia in the CFAS population-derived neuropathology cohort

**DOI:** 10.1016/j.neures.2024.01.003

**Published:** 2024-07

**Authors:** Hemant Mistry, Connor D. Richardson, Adrian Higginbottom, Bridget Ashford, Saif U. Ahamed, Zoe Moore, Fiona E. Matthews, Carol Brayne, Julie E. Simpson, Stephen B. Wharton, on behalf of the Cognitive Function and Ageing Study

**Affiliations:** a Sheffield Institute for Translational Neuroscience, and the Neuroscience Institute, the University of Sheffield, UK; b Populatiion Health Sciences Institute, Newcastle University, UK; c Cambridge Public Health, University of Cambridge, UK

**Keywords:** Cholesterol, HMG-CoA reductase, Sterol regulatory element-binding proteins, Dementia, Alzheimer’s disease, Cognitive Function and Ageing Study

## Abstract

Altered cholesterol metabolism is implicated in brain ageing and Alzheimer’s disease. We examined whether key genes regulating cholesterol metabolism and levels of brain cholesterol are altered in dementia and Alzheimer’s disease neuropathological change (ADNC). Temporal cortex (n = 99) was obtained from the Cognitive Function and Ageing Study. Expression of the cholesterol biosynthesis rate-limiting enzyme HMG-CoA reductase (HMGCR) and its regulator, SREBP2, were detected using immunohistochemistry. Expression of *HMGCR*, *SREBP2*, *CYP46A1* and *ABCA1* were quantified by qPCR in samples enriched for astrocyte and neuronal RNA following laser-capture microdissection. Total cortical cholesterol was measured using the Amplex Red assay. HMGCR and SREBP2 proteins were predominantly expressed in pyramidal neurones, and in glia. Neuronal HMGCR did not vary with ADNC, oxidative stress, neuroinflammation or dementia status. Expression of *HMGCR* neuronal mRNA decreased with ADNC (p = 0.022) and increased with neuronal DNA damage (p = 0.049), whilst *SREBP2* increased with ADNC (p = 0.005). High or moderate tertiles for cholesterol levels were associated with increased dementia risk (OR 1.44, 1.58). *APOE ε4* allele was not associated with cortical cholesterol levels. ADNC is associated with gene expression changes that may impair cholesterol biosynthesis in neurones but not astrocytes, whilst levels of cortical cholesterol show a weak relationship to dementia status.

## Highlights

• Cholesterol and its enzymes assessed in the CFAS neuropathology cohort.
• HMGCR protein is detected in cortical pyramidal neurones.
• HMGCR and SREBP2 mRNA expression alter with Alzheimer’s pathology.
• Higher levels of cortical cholesterol show a weak relationship to dementia status.
• Possession of *APOE ε4* allele does not affect cholesterol levels.

## 1 Introduction

Approximately 23% of the total cholesterol within the body is found within the brain, of which 70–80% is associated with myelination (Dietschy, 2009, Vitali et al., 2014). Cholesterol is essential for dendrite and synapse formation, and axonal guidance, whilst depletion of neuronal cholesterol leads to impairment of exocytosis of synaptic vesicles, neurotransmission and the degeneration of dendritic spines (Zhang and Liu, 2015). The potential role of cholesterol in brain ageing and neurodegenerative diseases is therefore an important question.

As the brain cannot absorb cholesterol from the blood due to the blood brain barrier (BBB), it meets its cholesterol demand by *de novo* biosynthesis (Bjorkhem and Meaney, 2004, Zhang and Liu, 2015). *C*holesterol biosynthesis occurs within the peroxisome and the endoplasmic reticulum (ER). The conversion of β-hydroxy β-methyl glutaryl Co A (HMG-CoA) to mevalonate, catalysed by HMG CoA reductase (HMGCR) is the rate-limiting step for the cholesterol biosynthesis pathway. Downstream steps can occur via two biochemical routes, the Bloch or Kandutsch-Russell pathways (Mazein et al., 2013). A hybrid of both of these pathways is used in the brain (Mitsche et al., 2015) (pathways are summarised in Appendix: Supplementary Figure 1). Cholesterol homeostasis involves transcriptional regulation of cholesterol biosynthesis genes by sterol regulatory element-binding proteins (SREBPs), and translocation of SREBP2 to the nucleus activates transcription of *HMGCR* to increase cholesterol biosynthesis (Goldstein et al., 2006, Simons and Ikonen, 2000, Sun et al., 2015).

Most studies have implicated astrocytes in cholesterol biosynthesis for post-mitotic neurones, with transport via APOE apolipoproteins (Pfrieger, 2003, Pfrieger and Ungerer, 2011, Zhang and Liu, 2015). Neurones possess receptors for APOE (LRP1) and disruption to the APOE system leads to an imbalance in cholesterol homeostasis within neurones (Pfrieger and Ungerer, 2011, van den Kommer et al., 2012). However, oxidative stress increases expression of HMGCR in neuronal cells, indicating that neurones are also capable of synthesising cholesterol (Clement et al., 2009).

A major excretion route of cholesterol from the brain is via conversion to oxysterols such as 24S-hydroxycholesterol (24S-OHC) and 27-hydroxycholesterol (27-OHC) which can pass through the blood brain barrier (BBB) (Chang et al., 2017, Sun et al., 2015). The conversion of cholesterol into 24S-OHC is catalysed by cholesterol 24-hydroxylase encoded by *CYP46A1* (Lund et al., 1999), and into 27-OHC by sterol 27-hydroxylase encoded by *CYP27A* (Heverin et al., 2005). Elevated levels of 24-OHC increase the production of free radicals, which leads to oxidative stress (Gamba et al., 2015, Kolsch et al., 2001).

Various studies suggest that cholesterol plays a complex role in Alzheimer’s Disease (AD). Excess brain cholesterol is linked to an increase in amyloid production (Hughes et al., 2013), whereby hypercholesterolemia increases cleavage of amyloid precursor protein (APP) by γ-secretase of the C99 terminal to release the Aβ polypeptide (Fassbender et al., 2002). APP may in turn regulate cholesterol turnover within neurones (Pierrot et al., 2013). A reduction in cholesterol synthesis due to a decrease in expression of 3β-hydroxysterol delta 24-reductase may underlie the imbalance in cholesterol in AD (Greeve et al., 2000, Martin et al., 2010, Waterham et al., 2001). Possession of the *APOE* ε4 allele is associated with decreased cholesterol delivery from glia to neurones (Gong et al., 2002, Martin et al., 2010). 24-hydroxylase is present in neuritic plaques, it is deficient in neurones making them more susceptible to stress, and its expression is increased in the presence of oxidative stress (Brown et al., 2004, Ohyama et al., 2006). The levels of 24-OHC are also decreased in AD brains, whilst 24-OHC favours the non-amyloidogenic processing of APP (Heverin et al., 2004, Martin et al., 2010, Sparks et al., 1994).

The aim of *this* study was to investigate how expression of key regulators of cholesterol biosynthesis (HMGCR and SREBP2), and of levels of cholesterol, vary in the ageing brain neocortex and how the expression of these varies with dementia status, Alzheimer’s disease neuropathological change (ADNC), DNA damage, and neuroinflammation. We used post-mortem human tissue from the Cognitive Function and Ageing Study (CFAS), a prospective population-representative longitudinal study of dementia and frailty that is representative of the UK population aged 65 yrs and above (Wharton et al., 2011). The population-representative nature of the neuropathology cohort allows assessment of the variation in cholesterol and associated biosynthetic enzymes in the ageing brain and their relationship to dementia status and ADNC without the biases inherent in preselected clinicopathological groups. The cohort design has been used in multiple CFAS studies (and other population/community-based cohort studies) (Nelson et al., 2022, Zaccai et al., 2006) and provides complementary insights to case-control studies. Previous studies in CFAS showed that oxidative DNA damage is associated with lower cognitive measures at early stages of ADNC (Simpson et al., 2015), and transcriptomic analysis demonstrated alterations in levels of RNA for genes on the cholesterol pathway, including *HMGCR* and *SREBF2* in neurones from cases with high levels of DNA damage (Simpson et al., 2016). This study also found an association between the neuronal DNA damage response and levels of 24(S)-OHC in the cerebrospinal fluid (CSF), which negatively correlated with increasing Braak neurofibrillary tangle (NFT) stage.

## 2 Methods

### 2.1 The CFAS and ageing brain cohort

Post-mortem human brain tissue was obtained from the Cambridge sub-cohort of CFAS (n = 99) as a population-representative sample. One hemisphere was sampled and fixed using formalin and stored at room temperature (RT). The other hemisphere was sliced in the coronal plane, snap frozen and stored at −80 °C. For both frozen and formalin-fixed paraffin-embedded (FFPE) sections the temporal cortex (Brodmann area 20/21) was investigated. Cohort demographics are show in Table 1. Dementia status at death was established as present, absent or uncertain, using a well-validated algorithmic approach using the AGECAT algorithm, death certification and a retrospective informant interview (Savva et al., 2009, Wharton et al., 2011). Donations were assessed for ADNC previously by neuropathologists, blinded to clinical information, as part of the CFAS study (Wharton et al., 2011). ADNC was assessed by a modified Consortium to Establish a Registry for Alzheimer’s Disease (CERAD) protocol, Braak neurofibrillary tangle (NFT) stage and Thal Aβ phase (Braak et al., 2006, Mirra et al., 1991, Thal et al., 2002). NFTs and plaques were semi-quantified in the temporal cortex as none, mild, moderate and severe according to CERAD criteria. The distribution of numbers of donations in Braak NFT stages was as follows: stages 0-II (entorhinal), 30; stages III-IV (limbic), 51; V-VI (isocortical), 18. The distribution of numbers of donations in each Thal Aβ phase was: phase 0, 13; phase 1, 9; phase 2, 17; phase 3, 24; phase 4, 19; phase 5, 17. The study received approval from the CFAS Management Committee and ethics committee approval (15/SW/0246). All tissue-based experiments were performed blinded to the dementia status of the donors.

**Table 1:** CFAS sample demographics.

Sample Demographics	Men (n = 37)	Women (n = 60)	Total (n = 98)
Age at death (years)	37	61	98
<80	10	11	21
80-89	19	28	47
>90	8	22	30
Mean age at death (years)	83.41	87.07	85.68
Median post-mortem delay (hours)	1.78	1.21	1.41
Median brain pH	6.43	6.52	6.49
missing (n)	7	11	18
Median cholesterol (n = 60)^a^	16.78	12.54	14.08
missing (n)	12	24	36
Median HMGCR	4.00	3.36	3.92
Dementia Status (n = 96) ^			
Dementia	16	22	38
No dementia	19	39	58
missing	2	0	2

a 62 samples with cholesterol measured with 2 outliers removed. Dementia status unknown for 2 samples.

### 2.2 Immunohistochemistry

Immunohistochemistry was carried out on FFPE tissue using a standard horseradish peroxidase-conjugated avidin-biotin complex (ABC-HRP) technique with 3,3’-diaminobenzidine (DAB) as substrate (Vector laboratories, UK). The conditions for antigen retrieval and antibody details are provided in Table 2. Sections were counterstained in Harris’s haematoxylin, then blued in Scotts tap water. Rabbit isotype controls and omission of primary antibody were included in every run as negative controls. Assessment of average percentage specific immunoreactivity was carried out by capturing brightfield microscopic images across two continuous non-overlapping belts across all layers of the cortex, using a x20 objective (Nikon Eclipse Ni-U microscope, Nikon, UK) and quantified using the Analysis^D software (Olympus Biosystems, UK).

**Table 2:** Antibodies used for Immunohistochemistry.

Antibody (catalogue number)	Species, optimised dilution and incubation conditions	Antigen retrieval
HMGCR (Abcam, UK, ab174830)	Rabbit monoclonal, 1:100, overnight 4 C	Pressure cooker 1 hr, pH9.5 buffer
HMGCR (Atlas Antibodies, Sweden, AMAb90619)	Mouse monoclonal, 1:100, overnight 4 C	Pressure cooker 1 hr, pH9.5 buffer
SREBF2 (Abcam, UK, ab28482)	Rabbit IgG polyclonal, 1:100, 1 hr at RT	Microwave 10 mins, pH6 buffer
GFAP (DAKO, Denmark, Z033401-2),	Rabbit IgG polyclonal, 1:500, overnight 4 C	n/a

### 2.3 Quantification of neurones in temporal cortex

Formalin-fixed paraffin embedded (FFPE) temporal cortex blocks were sectioned at 5 µm and stained with haematoxylin and eosin (H&E). Stained sections were digitally scanned under a 40x objective using the NanoZoomer XR (Hamamatsu, Photonics Ltd., Hertfordshire, UK). Digitised slide images were stored as NanoZoomer Digital Pathology Image (.ndpi) files, viewed and exported using NDP.View2Plus. Counts of neuronal pyramidal cells were quantified by taking non-overlapping images in three adjacent belt transects encompassing the cortical thickness at 20x magnification. Cell count analysis was performed using Fiji software (Schindelin et al., 2012). Briefly, the colour deconvolution extension was used to identify blue nuclei. The contrast of all images increased to better visualise nuclei and the image was thresholded. Total cell counts were taken. Pyramidal neurone nuclei counts were taken using a size exclusion criteria of > 260 pixels (Simpson et al., 2015). Neuronal cell counts were expressed as average number of cells per field area (field area 0.384 mm^2^). For these analyses, suitable H and Es were available for 93 of our cohort.

### 2.4 Western blotting

Western blotting was carried out to validate detection of HMGCR by the Abcam antibody on protein extracts from a sample of frozen temporal cortex and from cell lines, including HeLa cells, Lund Human Mesencephalic Cells (LUHMES, ATCC CRL-2927^TM^)) differentiated human neuronal cells and human primary fetal astrocytes (ScienCell Research Laboratories, Carlsbad CA, US) (Ratcliffe et al., 2018, Vazquez-Villasenor et al., 2021). Protein was extracted by homogenisation in Tris extraction buffer (10 mM Tris–HCl pH 7.4, 0.8 M sodium chloride, 1 mM EDTA, 10% sucrose, 0.1mMPMSF, 2 µg/ml aprotinin, 10 µg/ml leupetin, 5 µg/ml pepstatin, 40 mM β-glycerophosphate, 50 mM sodium fluoride, 200 µM sodium orthovanadate), and centrifuged at 14,000 rpm at 4 °C for 30 mins. The cell pellets were lysed in ice-cold extra strong lysis buffer (100 mM Tris-HCl (pH 7.5), 0.5% (w/v) sodium dodecyl sulfate (SDS), 0.5% (w/v) sodium deoxycholate, 1% (v/v) Triton X-100, 75 mM sodium chloride (NaCl), 10 mM ethylenediaminetetraacetric acid, 2 mM sodium orthovanadate, 1.25 mM sodium fluoride, protease inhibitor cocktail and PhosStop (both Roche, Basel, Switzerland) followed by centrifugation at 16,000 g at 4 °C for 5 min. Protein content of samples was measured using bicinchoninic acid method and equal amounts (30 µg) analysed by Western blot analysis and transferred onto nitrocellulose membranes (GE Healthcare, UK). The membrane was blocked for non-specific binding in 5% milk in PBS-T for 1 hr at RT, followed by a primary antibody (Table 3) incubation at 4 °C overnight. The membranes were washed with 1x PBS-T followed by species-specific HRP conjugated secondary antibody incubation for 1 hr at RT. To confirm equal protein loading, the membrane was reprobed for α-tubulin. Protein expression levels were determined by densitometry of the appropriate, subsaturated band using the G-box Chemi-XT CCD Gel imaging system (Syngene, UK) and the results normalized to α-tubulin.

**Table 3:** Antibodies used for Western blotting.

Antibody (catalogue number)	Species, dilution, incubation conditions
HMGCR (Abcam, UK, ab174830)	Rabbit monoclonal, 1:1000, overnight 4 C
α-tubulin (Sigma Aldrich, UK, clone DM1A, T6199)	Mouse monoclonal, 1:1000, overnight 4 C
Anti-rabbit HRP (Promega, UK, W4011)	Goat polyclonal, 1:5000, 1 hr RT
Anti-mouse HRP (Promega, UK, W4021)	Goat polyclonal, 1:5000, 1 hr RT

### 2.5 Laser capture microdissection

Frozen tissue of temporal cortex was used from a sub-group of cases (n = 32), selected based on pH and post-mortem delay, and stratified by Braak NFT stage to include 12 from entorhinal stages (0-II), 10 from limbic stages (III-IV) and 10 from isocortical stages (V-VI). Freshly prepared frozen cryosections (7 µm) were collected onto uncharged glass slides, fixed and permeabilised in ice cold acetone (VWR Chemicals, France) for 3 min. To visualise neurones, sections were immersed in toluidine blue for 1 min. To visualise astrocytes, rapid avidin-biotinylated complex-horse radish peroxidase (ABC-HRP) immunostaining for GFAP (Dako, Denmark [Z033401–2], used at 1:50) was carried out as described previously (Waller et al., 2012). The sections were dehydrated in a graded series of ethanol, extensively cleared in xylene and air dried for 1 hr in a flow hood. LCM was performed using Pixcell laser capture microdissection system (Arcturus, Thermofisher scientific, UK) at x20 magnification to micro-dissect approximately 1000 GFAP^+^ astrocytes and 1000 neurones per case (laser power: 55 mW, laser pulse: 15 ms and laser spot size: 7.5 µm) to obtain approximately 50 ng of RNA. The thermoplastic film was carefully removed from the cap using sterile forceps and placed in a 0.5 ml sterile tube for RNA extraction. Sterile solutions made with diethylpyrocarbonate (DEPC)‐treated water and RNAse‐free conditions were used throughout this protocol. Cell enrichment was assessed for each sample by RT-PCR analysis. All astrocyte samples showed a strong band for GFAP at 213 bp while neuronal samples showed a strong band for NeuN at 127 bp. β-actin was used as a positive control for both samples. A non-template control containing no cDNA was used as a negative control.

### 2.6 RNA extraction

Total RNA was extracted using the Picopure RNA isolation kit (Thermo fisher scientific, UK) according to the manufacturer’s instructions. The RNA concentration of each sample was determined using the NanoDrop Spectrophotometer (ThermoFisher Scientific, USA) and RNA integrity was determined using the Agilent RNA 6000 Pico Chip (Agilent Technologies, USA).

### 2.7 Quantitative real time PCR (qPCR)

Expression levels of *HMGCR*, *CYP46A1*, *ABCA1* and *SREBP2* were assessed by qPCR. Each RNA sample was synthesised into cDNA using qScript cDNA mix (Quanta biosciences, USA), according to the manufacturer’s protocol using a Peltier thermocycler (MJ Research, USA), and analysed using PrimeTime qPCR assays (Integrated DNA Technologies) (Table 4). PCR was performed using 50 ng cDNA, 500 nM of forward and reverse primer, 250 nM probe and 1x LUNA universal probe qPCR master mix (New England Biolabs, UK) in a total volume of 10 μl per sample. Each sample was assessed in triplicate. Following denaturation at 95 °C for 10 min the products were amplified (40 cycles at 95 °C for 30 s and 60 °C for 60 s) using the BioRad CFX384 Real time system. β-actin was also amplified on each plate to normalise the gene expression levels using the comparative Ct, (ΔΔCt) method of quantification.

**Table 4:** List of Prime Time® qPCR Assays for cholesterol biosynthetic gene expression.

Gene	Prime Time Assay ID	Reference	Exon	Primer sequence	Probe sequence
*HMGCR*	HS.PT5 8.4048748	NM_001130996	15-16	1) 5’-CCTTTATATCCGTTTCCAGTCCA-3’ 2) 5’-CCACTAACGGCTAGAATCTGC-3’	5’-/56-FAM/ATGTTCATC/ZEN/CCCATGGCATCCCC/3IABkFQ/−3’
*CYP46A1*	Hs.PT.58.26445892	NM_006668	12-13	1) 5’-AAGAGTCGCTGAGGCTGTA-3’ 2) 5’-CCTCAAAGTATGTGTCCATCCG-3’	5’-/56-FAM/CTCCTCTTC/ZEN/CAGCAGGCGAAAGG/3IABkFQ/−3’
*ABCA1*	Hs.PT.58.11955	NM_005502	49-50	1) 5’-TGCTACAATACCAGCTTCCATC-3’ 2) 5’-GTCCTTGGCAAAGTTCACAA-3’	5’−56-FAM/TCTCCCAGA/ZEN/GCAAAAAGCGACTCC/3IABkFQ/−3’
*SREBF2*	Hs.PT.58.39417166	NM_004599	16-17	1) 5’-TTCCTTCTGCCATTGCGA-3’ 2) 5’-ACAGTAGCAGGTCACAGGT-3’	5’−56-FAM/CTATGGAGC/ZEN/AGCCTCAACGTCAGT/3IABkFQ/−3’
*ACTB* (Beta-actin)	Hs.PT.39a.22214847	NM_001101	1-2	1) 5’-ACAGAGCCTCGCCTTTG-3’ 2) 5’-CCTTGCACATGCCGGAG-3’	5’−56-FAM/TCATCCATG/ZEN/GTGAGCTGGCGG/3IABkFQ/−3’

### 2.8 Measurement of cholesterol

Freshly prepared cryosections (10 µm) from all available donations with suitable frozen temporal cortex tissue from the Cambridge cohort (n = 63) were homogenised and analysed using the Amplex Red cholesterol assay according to manufacturer’s protocol (Thermo fisher scientific, UK). Each sample was run in triplicate, the excitation fluorescence was measured at 540 nm and the emission was measured at 590 nm using the PHERAStar spectrophotometer. Background fluorescence was corrected for each well and the cholesterol concentration calculated from the standard curve. Cholesterol concentrations were divided by the mass of tissue used to give concentration per mg of tissue.

### 2.9 Statistical Analysis

Statistical analysis was carried out using SPSS Statistics v26 (IBM, UK), STATA version 15 (StataCorp 2017 Stata Statistical Software: Release 15. College Station, TX: StataCorp LLC, USA), and R (RStudio Team 2020. RStudio: Integrated development for R. RStudio, PBC, Boston MA URL http://www.rstudio.com/). For plotting ggplot2 was used (H.Wickham. ggplot2: Elegant Graphics for Data Analysis. Springer-Verlag New York 2016). Data were summarised using mean, standard deviation (SD), median and interquartile range (IQR). A Kolmogorov Smirnov test was carried out to determine if the data were normally distributed. Mann-Whitney U-test was used to determined quantitative differences between groups, whilst the Kruskal-Wallis (KW) and Jonckheere-Terpstra (JT) tests were performed to determine significant differences and trends respectively in the case of more than two groups. Correlation was assessed using Spearman rank correlation. Mann-Whitney U-test was used to determine differences between those with and without dementia at death. Variations in measures between those with and without dementia were visualised using violin plots and the effect of measures on risk of dementia was analysed using logistic regression and generalized linear regression models. ANC was measured using Braak NFT staging and Thal β-Amyloid phase. For statistical analysis, Braak NFT stages were combined to allow comparisons between Braak groups and Thal phases were also grouped to allow for comparison between Thal phases. Groups were defined as follows: for Braak, group 1 contained stages 0-II (entorhinal), group 2 contained stages III-IV (limbic) and group 3 contained stages V-VI (isocortical); for Thal, group 1 contained phases 1–2, group 2 contained phases 3–4 and group 3 contained phases 5–6. Local measures of ADNC in the temporal cortex included CERAD plaque and NFT scores. 24(S)-OHC levels in CSF, oxidative stress (ɣH2AX, 8-hydroxydeoxyguanosine [8-OHdG] and malondialdehyde expression), β-amyloid, phosphorylated tau expression (AT8 antibody), brain pH, neuroinflammation (CD68, GFAP and MHC-II), and post-mortem delay (PMD) in the cases were obtained from previous studies (Simpson et al., 2010a, Simpson et al., 2010b). Tests were two tailed and p < 0.05 was set as the level for significance.

## 3 Results

### 3.1 HMGCR and SREBP2 expression is predominantly associated with neurones in the temporal cortex of the ageing brain

**Fig. 1:**
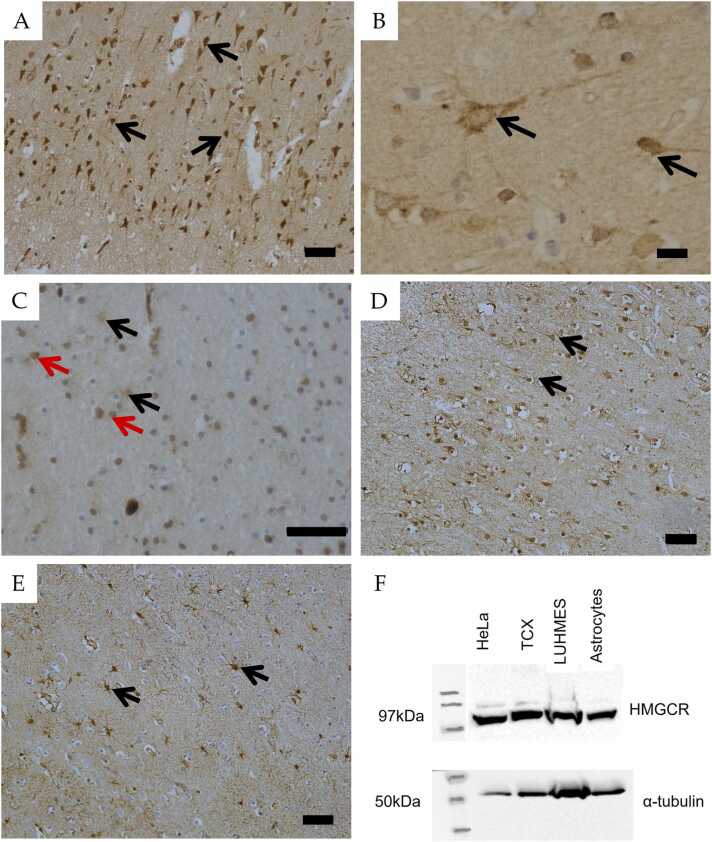
Histological characterization of the cholesterol biosynthesis pathway in the human brain. HMGCR expression was associated with pyramidal neurons throughout all layers of the temporal cortex (A, black arrows). HMGCR immunoreactivity was predominantly detected in the cytoplasm of neuronal cell bodies and proximal processes (B, black arrows). Immunopositive glial cells were detected in approximately 35% (n = 35) of the cohort, associated with cell with astrocyte-like morphology (C, black arrows) and other glia (C, red arrows). SREBP2 immunoreactivity was associated with pyramidal neurons within the temporal cortex (D) and in astrocytes within the white matter and grey matter border regions of the temporal cortex (E). Western blot for the HMGCR antibody (Abcam, UK) for HeLa cells, post-mortem human temporal cortex (TCX) protein extract, LUHMES, astrocyte lysates (F). Scale bar represents 50 µm.

Expression of HMGCR was punctate and localised to the neuronal soma and proximal processes (Fig. 1 A and B). Positive neuronal expression of HMGCR was confirmed in frozen temporal cortex (Appendix: Supplementary Figure 2). HMGCR expression was also associated with cells morphologically resembling glia, including astrocytes and oligodendrocytes within the cortex and white matter border of approximately 35% of cases (n = 35), (Fig. 1 C). Western blotting identified a 97 kDa band in both tissue and cell extracts, confirming that the antibody identified HMGCR at its predicted molecular weight (Kumar et al., 2019) (Fig. 1 F).

Neuronal expression of SREBP2, the key regulator of HMGCR, was localised to the cytoplasm and proximal process within cortical regions. At the cortical and white matter border SREBP2 immunoreactivity was associated with the cell body & processes of cells morphologically resembling astrocytes (Fig. 1 D and E).

### 3.2 Neuronal HMGCR expression does not relate to ADNC, oxidative stress, neuroinflammation or dementia status

**Fig. 2:**
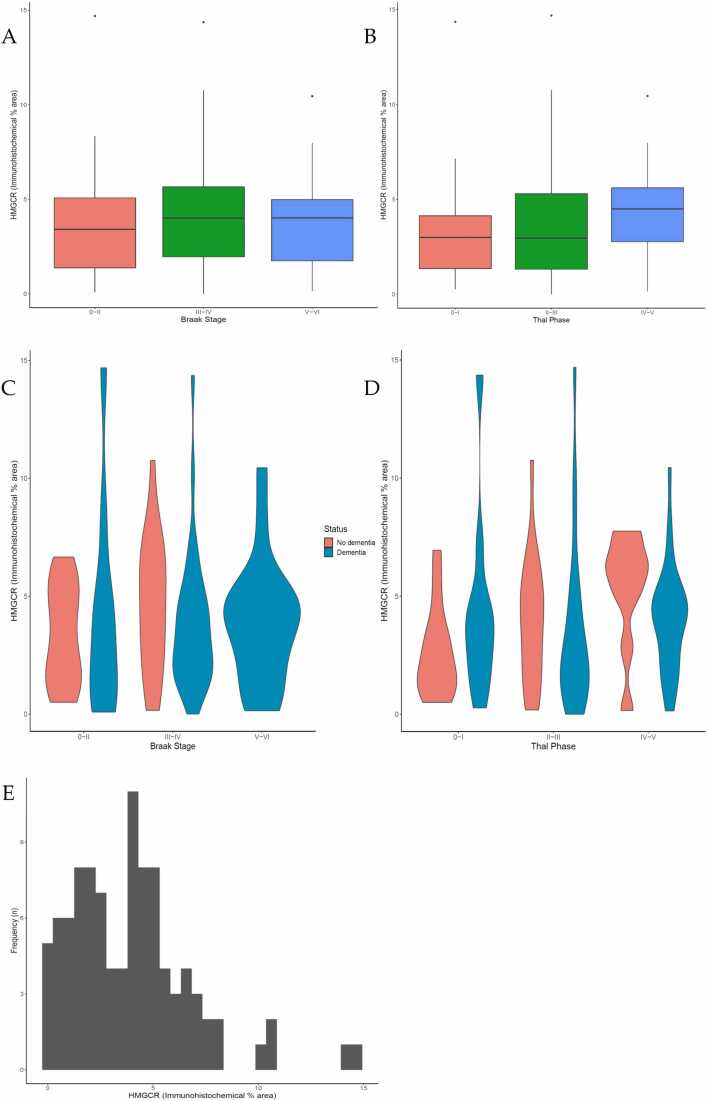
HMGCR immunoreactivity does not vary with AD pathology markers or dementia status in the human brain. Outliers in boxplot A and B shown as circles. Boxplots of the percentage area immunoreactivity of HMGCR versus Braak neurofibrillary tangle stage (A) and Thal Aβ phase (B). For both Braak and Thal staging, groups have been combined (see Methods). Violin plots showing the distribution of HMGCR immunoreactivity by dementia status at specific Braak stage (C) and Thal phase (D). No significant difference HMGCR immunoreactivity between individuals with and without dementia was found within Braak stages and Thal phases. Histogram shows the distribution of the variation in percentage area of immunoreactivity of HMGCR across the cohort (E).

Expression of HMGCR, as the rate-limiting enzyme of cholesterol biosynthesis, was then quantified as percentage area expression using image analysis to determine whether expression varied with neuropathological measures and dementia status at death. The variation in HMGCR % immuno-expression in the cohort was positively skewed (Kolmogorov-Smirnov p = 0.092; mean 3.90%, SD 2.86, median 3.87, IQR 1.69–5.26). HMGCR neuronal expression did not vary with Braak NFT stage (KW p = 0.559; JT p = 0.578) (Fig. 2 A), nor with Thal Aβ phase (KW p = 0.311; JT p = 0.171) (Fig. 2 B). Variation in HMGCR immunoreactivity was also assessed according to local measures of ADNC in temporal cortex. No significant correlations were found between neuronal HMGCR immunoreactivity and either Aβ percentage immunoreactivity (r = −0.027, p = 0.800), or phosphorylated tau percentage immunoreactivity (r = −0.083, p = 0.434). Examination of serially stained sections showed expression of HMGCR in neurones irrespective of the presence of AT8-positive NFT (Appendix: Supplementary Figure 3), although more detailed quantitative double-labelling studies would be required to address the effects of tau pathology on HMGCR expression in individual neurones. To obtain a correction for neuronal numbers (which can vary with Alzheimer’s progression) HMGCR % immuno-expression values were divided by pyramidal neurone counts. Statistical tests were repeated but these remained non-significant.

Data from our previous studies were used to study correlations with HMGCR immunoreactivity and markers of DNA damage (γH2Ax), oxidative stress (malondialdehyde) and neuroinflammation (Simpson et al., 2010a, Simpson et al., 2010b). HMGCR immunoreactivity did not correlate with γH2Ax immunoreactivity in either neurones (r = −0.039, p = 0.706) or astrocytes (r = 0.060, p = 0.563), or protein levels of malondialdehyde (r = −0.008, p = 0.966). HMGCR immunoreactivity did not correlate with the neuroinflammation markers CD68 (r = −0.034, p = 0.752) and MHC-II (r = −0.055, p = 0.617) as measures of microglial activation.

The distribution of HMGCR immunoreactivity by dementia status across specific Braak NFT stages and Thal Aβ phases was determined (Fig. 2 C and D). A pairwise comparison of means using Tukey’s adjustment found no significant difference between HMGCR immunoreactivity and dementia status at specific Braak NFT stages and Thal Aβ phases. The effect of HMGCR immunoreactivity on risk of dementia was analysed using logistic regression (Table 5). A univariate model adjusted for only age at death and sex as covariates, as well as a multivariate model adjusted for Braak, Thal and CAA pathology was produced. HMGCR immunoreactivity was analysed as continuous scales and categorically divided into tertiles. None of HMGCR models showed any effect on risk of dementia. To ensure that the variation in immunostaining was not an artifact of brain collection procedures or pre-mortem status, correlations between brain pH (r = 0.094, p = 0.405) and post-mortem delay (r = −0.026, p = 0.813) were determined; no significant correlations were found.

**Table 5:** Logistic regression modelling for HMGCR immunoreactivity and dementia status.

	Unadjusted (univariate)			Adjusted (multivariate)		
	OR	95% CI	p	OR	95% CI	p
HMGCR	0.96	0.82-1.12	0.58	0.96	0.82-1.13	0.94
Tertiles						
Moderate	0.81	0.26-2.53	0.72	0.69	0.2-2.47	0.72
High	0.47	0.15-1.4	0.18	0.4	0.11-1.42	0.18

This table shows the output from logistic regression modelling. A univariate model adjusted for age at death and sex, multivariate models adjusted for Braak, Thal and CAA pathology. HMGCR immunoreactivity was modeled on a continuous scale and categorically by tertiles. Results are presented as odd ratios (OR) with 95% confidence intervals (CI) and p values.

### 3.3 *HMGCR* and *SREBP2* alterations with ADNC, DNA damage and neuroinflammation

**Fig. 3:**
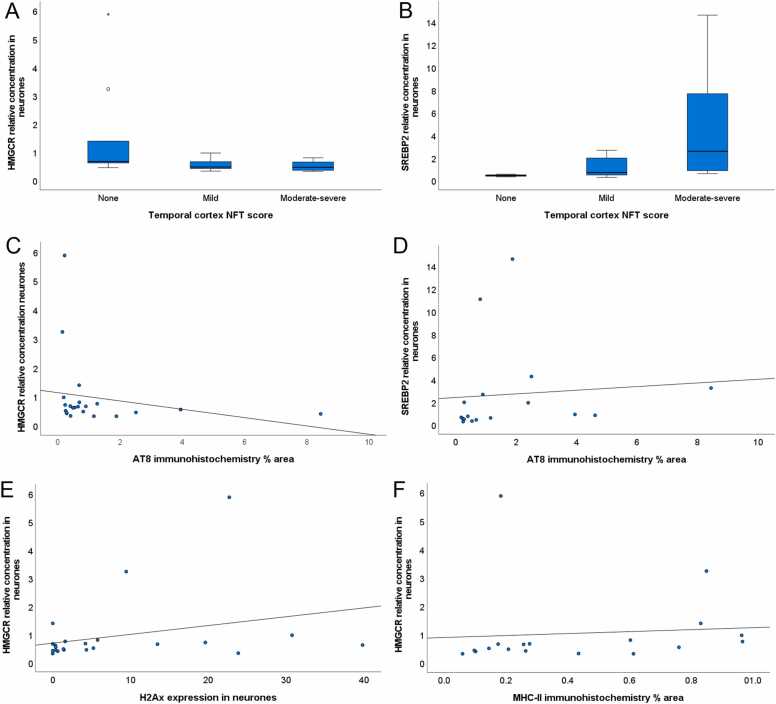
Cholesterol biosynthetic gene expression is altered with NFT pathology markers in the temporal cortex. Box plots showing the distribution of relative RNA concentration (y-axis) for *HMGCR,* and *SREBP2* respectively across CERAD neurofibrillary tangle (NFT) scores, with moderate to severe combined (x-axis) (A-B). Outliers in boxplot A shown as circle and asterisk. *HMGCR* expression in neurons suggests a significant trend to decrease as NFT score increases (JT p = 0.022) (A). *SREBP2* expression in neurons suggests a significant difference and trend to increase as NFT score increases (KW p = 0.022; JT p = 0.005) (B). Scatter plots showing the neuronal relative concentration (y-axis) for *HMGCR* (r = −0.458; p = 0.032) and *SREBP2* (r = 0.574; p = 0.016) respectively against AT8 immunoreactivity (x-axis) (C-D). Scatterplot showing the neuronal relative concentration (y-axis) for *HMGCR* and neuronal γ (r = 0.419, p = 0.046) (E) and for *HMGCR* and MHC class II (r = 0.490, p = 0.039). Relative concentration values for each gene were calculated relative to Braak Group 1. 0 = no tangles, 1 = mild tangles 3 =moderate-severe tangles.

To determine if expression of cholesterol biosynthetic genes in neurones varied with ADNC, we examined the relationship of mRNA levels of these genes to local measures of ADNC in the temporal cortex. *HMGCR* expression in neurones significantly decreased (KW; p = 0.59, JT; p = 0.022) as local temporal cortex NFT score (Fig. 3A) and neuritic plaque scores increased (KW; p = 0.022, JT; p = 0.020). However, they did not show alterations with Braak NFT stage or with Thal Aβ phase. A significant difference (KW; p = 0.022) and a trend to increase (JT; p = 0.005) was observed between *SREBP2* relative concentration and NFT score (Fig. 3B) and also with Braak NFT stage (KW; p = 0.046, JT; p = 0.011), but this did not alter with either neuritic plaque score or Thal Aβ phase. For astrocytes, *SREBP2* decreased with Braak NFT stage (JT; p = 0.031) but there were no other significant alterations. To determine whether these relationships were affected by neuronal number, we divided the respective mRNA measures by pyramidal neurone counts. The increase of SREBP2 mRNA relative concentration with local measures of ADNC was maintained, with a significant increase with temporal cortex NFT score (JT; p = 0.035). Whilst there still appeared to be a trend to increased with CERAD neuritic plaque score, this did not reach significance (JT; p = 0.066). As for the measures uncorrected by neuronal number, SREBP2 expression did not change with Braak NFT stage or Thal phase. For HMGCR, although the medians tended to decrease with increasing ADNC, the results no longer reached statistical significance when mRNA values were divided by neuronal cell counts (Appendix: Supplementary Figure 4).

We also examined the relationship to % area AT8 immunoreactivity in the temporal cortex. The relative concentration of *HMGCR* in neurones showed a moderate negative correlation with AT8 immunoreactivity (r = −0.458, p = 0.032) (Fig. 3C). The relative concentration of *SREBP2* in neurones showed a moderate positive correlation with AT8 immunoreactivity (r = 0.574, p = 0.016) (Fig. 3D). Expression in astrocytes did not show significant alterations.

We then examined whether *HMGCR* and *SREPBP* mRNA expression altered in response to oxidative stress by examining correlations with γH2Ax in astrocytes and neurones, malondialdehye and 8-OHdG. Relative concentration of *HMGCR* mRNA increased with neuronal γH2Ax (r = 0.419, p = 0.046) but did not show significant correlations with the other markers. *SREPBP2* expression did not alter with these markers. We examined the effect of neuroinflammation by correlation analysis with MHC class II, CD68 and GFAP. *HMGCR* increased with MHC class II (r = 0.490, p = 0.039), but did not show significant correlations with the other markers. *SREPBP2* expression did not alter with these neuroinflammation markers. Expression in astrocytes did not show significant alterations.

To assess genes involved in cholesterol transport and metabolism, we examined expression of *ABCA1* and *CYP46A* in neurones and astrocytes. These genes showed no significant changes in expression with markers of AD neuropathology, DNA damage or oxidative stress in either cell type.

### 3.4 Brain tissue cholesterol concentration relationship to dementia status

**Fig. 4:**
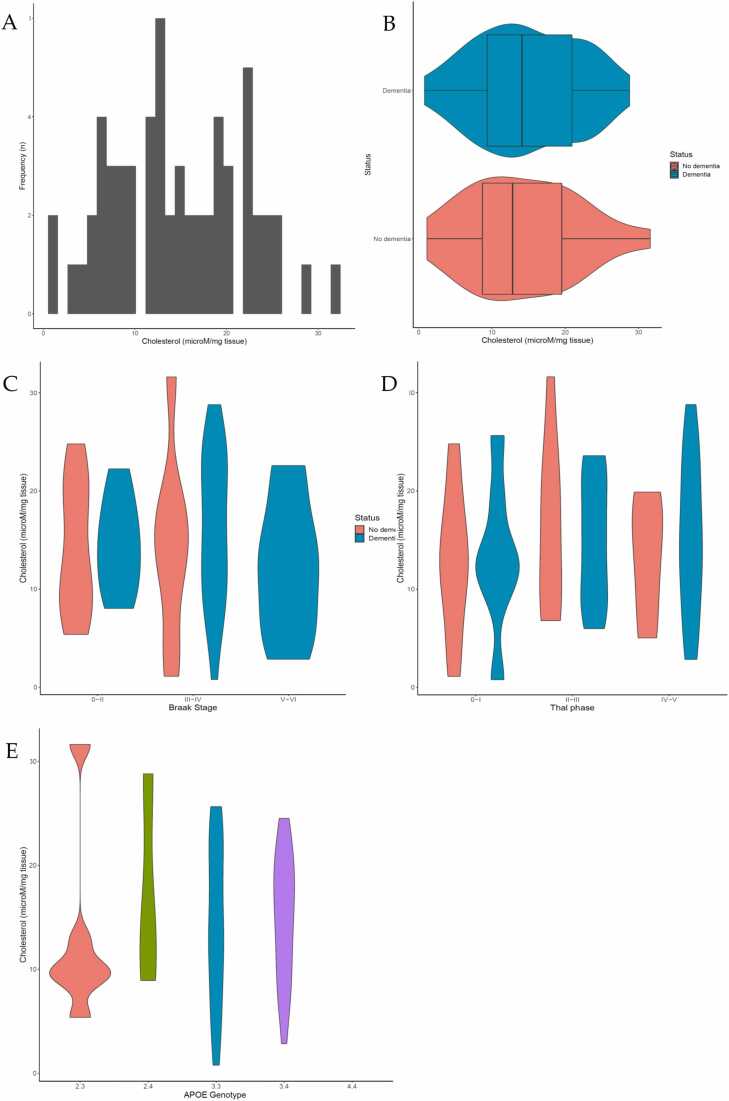
Brain Tissue cholesterol concentration. Histogram showing the variation in tissue cholesterol concentration across the cohort (A). Box and violin plots displaying the distribution and probability density of tissue cholesterol concentration by dementia status. Box plots show median, IQR and range, area around the box displays the probability density. (B). Violin plots showing the distribution of tissue cholesterol concentration by dementia status at specific Braak stage (C) and Thal phase (D). Violin plots showing the distribution of tissue cholesterol concentration by *APOE* genotype (E). Wider sections of the violin plots represent a higher probability that members of the population will take on the given value; narrower sections represent a lower probability. Figures represent measures with cholesterol outliers removed.

We then sought to directly measure how brain cholesterol concentration changed with pathology. Temporal cortex tissue cholesterol concentration was determined using the Amplex Red cholesterol assay. The distribution of cholesterol concentrations is shown in (Fig. 4A). Of the cases where cholesterol could be measured (n = 63), 2 were excluded from analysis as outliers. Of the 61 individuals remaining, mean cholesterol for the sample was 14.58 µM/mg (Standard error (SE) ± 7.14). Brain cholesterol concentrations showed only very weak correlations with tissue pH (r = 0.262, p = 0.041) and PMD (r = −0.278, p = 0.033).

Of the 61 individuals, 40 had a dementia diagnosis at death and 21 had no dementia (Fig. 4B). There was no significant difference in tissue cholesterol concentration between individuals with (median=14.08, IQR= 9.06–21.76) and without (median= 12.84, IQR= 8.37–20.14) dementia (MW, p = 0.95). The distribution of tissue cholesterol concentration by dementia status across specific Braak NFT stages and Thal Aβ phases was determined (Fig. 4 C and D). There was no significant difference in tissue cholesterol concentration between those with and without dementia at specific Braak NFT stages and Thal Aβ phases. The effect of tissue cholesterol concentration on risk of dementia was then analysed using logistic regression (Table 6). A univariate model adjusted for only age at death and sex as covariates, and a multivariate model adjusted for Braak NFT stage, Thal Aβ phase and cerebral amyloid angiopathy was produced. Tissue cholesterol concentration was analysed as continuous scales and we also modelled with data categorically divided into tertiles to identify a more clinically significant effect of being in the highest tertile compared to the lowest. When tissue cholesterol was divided into tertiles, point estimates showed increased risk of dementia in moderate (univariate OR: 1.58, 95% CI: 0.39–6.43; multivariate OR: 1.43 95% CI: 0.31–6.53) and high cholesterol groups (univariate OR:1.44, 95% CI: 0.35–5.85; multivariate OR: 1.21 95% CI: 0.29–5.08). In contrast the estimate for continuous cholesterol measure showed no effect on dementia status (OR: 0.98, 95% CI: 0.93–1.03).

**Table 6:** Logistic regression modelling of cholesterol concentration with dementia status.

	Unadjusted (univariate)			Adjusted (multivariate)		
	OR	95% CI	p	OR	95% CI	p
Cholesterol	0.98	0.93-1.03	0.39	0.98	0.93-1.03	0.33
Tertiles						
Moderate	1.58	0.39-6.43	0.52	1.43	0.31-6.53	0.65
High	1.44	0.35-5.85	0.61	1.21	0.29-5.08	0.80

Moderate and high tissue cholesterol concentration is associated with dementia status. Output from logistic regression modelling. A univariate model adjusted for age at death and sex, and multivariate models adjusted for Braak, Thal and CAA pathology. Tissue cholesterol concentration was modeled on a continuous scale and categorically by tertiles. Results are presented as odd ratios (OR) with 95% confidence intervals (CI) and p values.

*APOE*-genotype was available for 57 of the individuals. There were no significant differences between *APOE*-genotypes for cholesterol levels (p = 0.67) (Fig. 4E), nor did cholesterol levels differ between individuals with and without the ε4 allele (p = 0.76) (Appendix: Supplementary Table 1). Categorical analysis of cholesterol tertiles showed no significant effect between low, moderate, and high cholesterol concentrations and *APOE* status.

Statistical analyses of the relationship of cholesterol to oxidative stress, neuroinflammation and vascular pathology were performed and found no association with brain tissue cholesterol concentration (Appendix: Supplementary Table 2).

## 4 Discussion

We show that the two key regulators of cholesterol biosynthesis, HMGCR and SREBP2, are predominantly associated with pyramidal neurons in cortex, though some glial expression was also detected. Although protein expression did not vary with cellular pathologies, gene expression levels of *HMGCR* in neurons (but not astrocytes) decreased with several ADNC measures whilst, conversely, *SREBP2* increased in neurons and decreased in astrocytes. Increased levels of *HMGCR* also positively correlated with neuronal DNA damage. In contrast to its rate-limiting biosynthetic enzyme, higher cholesterol tertiles were associated with a higher risk of dementia.

HMGCR immunolabelling of pyramidal neurones was observed across Braak NFT stages in the CFAS cohort (of individuals aged 65 and over), so that the cellular expression pattern is not altered by ADNC. Western blotting in whole tissue and cell-culture protein extracts confirmed the specificity of the antibody for HMGCR, and its expression in neurones. Many studies have shown that glial cells undertake the bulk of the cholesterol production within the brain, whereas the current study suggests that neurones possess the mechanisms to synthesise cholesterol (Nieweg et al., 2009, Pfrieger, 2003, Pfrieger and Ungerer, 2011, Zhang and Liu, 2015). Astrocytes are considered the major source of neuronal cholesterol in vivo, which is transported to neurones via APOE. Astrocyte-derived rather than neuronal cholesterol is particularly important for synaptic function (Li et al., 2022). Neuronal expression of HMGCR suggests these cells can regulate their own homeostasis in human brain, and also highlights a need for a better understanding of the relative contributions of glial and neuronal cholesterol in health and disease, and the role of varying biosynthetic pathways.

One possible biological explanation for the lack of correlation between HMGCR expression and cholesterol concentration that we observed could be that HMGCR activity is used to produce isoprenoids rather than cholesterol (Eckert et al., 2009). Isoprenoids are involved in the prenylation of proteins, a post-translational modification allowing insertion into membranes. Isoprenoids are formed from the mevalonate arm of the cholesterol biosynthesis pathway in neurones and are important in neuronal functions (Govek et al., 2005, Hooff et al., 2008, Hooff et al., 2012, Moutinho et al., 2017). They may also be altered by ageing and neurodegeneration (Edlund et al., 1994, Mohamed et al., 2012), suggesting that further definition of these cholesterol-related metabolites in dementia is important.

In this study*, HMGCR* mRNA in neuronal populations decreased with increasing NFT pathology and there was a significant moderate negative correlation with phospho-tau immunoreactivity. In contrast, *SREBP2* in neuronal populations increased with Braak NFT stage, local cortical NFT pathology and AT8 immunoreactivity. These data suggest that the gene expression of key regulators of the cholesterol biosynthesis pathway (HMGCR and SREBP2) are altered in ADNC progression. It should be noted that, when we corrected for numbers of pyramidal neurones present, the increase of *SREBP2* expression with local ADNC measures (particularly NFT) was maintained whilst the relationship of *HMGCR* was attenuated. Neuronal density may change with ageing and neurodegeneration, so this may suggest that neuronal numbers and neuronal loss may complicate the analysis. However, it should be noted that the 2-dimensional assessment of neuronal numbers may not be accurate, introducing greater variability, and may introduce biases (e.g. through the effects of changes in cell size). Alternative stereological and/or biochemical measures are likely to be more accurate, and further work is required to determine how changes in neuronal number may truly affect expression levels.

The changes in gene expression in the enriched neuronal population were not translated to a change in brain tissue cholesterol concentration. The changes in *SREBP2* expression might suggest a compensatory mechanism in neurones, as cholesterol levels are not altered. As *SREBP2* expression shows a trend to increase it may later lead to an increase in expression of SREBP2 target genes such as *HMGCR* (Sharpe and Brown, 2013). Given the lack of *HMGCR* alteration in astrocytes and the fall of *SREBP2* with Braak NFT stage progression, we did not find evidence of a compensatory response in astrocytes, raising the question of whether astrocytes might fail to respond to changes in neuronal cholesterol biosynthesis gene expression with ADNC progression.

We also found that neuronal *HMGCR* expression showed a moderate positive correlation with oxidative DNA damage markers (γH2AX). We previously showed overexpression of *HMGCR* in neurones isolated from frontal cortex of low Braak NFT stage cases that had high DNA damage response (Simpson et al., 2016). Furthermore, exposing human neuroblastoma cells to oxidative stress also results in an increase in *HMGCR* expression (Recuero et al., 2009). Cholesterol influences the processing of APP, cholesterol enriched regions of the membrane favour the production of amyloidogenic Aβ, and APP has also been shown to inhibit cleavage of SREBP2, preventing the nuclear localisation of the transcription factor domain (Cordy et al., 2003, Grimm et al., 2012, Pierrot et al., 2013). The deposition of Aβ plaques is a source of oxidative stress leading to DNA damage within neurones (Kwiatkowski et al., 2016). This suggests that APP prevents the expression of SREBP2 target genes; however, the presence of Aβ plaques leads to the generation of reactive oxygen species (ROS) and oxidative stress, which may induce *HMGCR* expression. This may lead to the aberrant activation of cholesterol biosynthesis and cholesterol accumulation in cells, which could increase the generation of Aβ.

*This* study found that moderate and high levels of brain cholesterol are associated with increased risk of dementia at death. This association was observed in both univariate analysis, adjusted for age at death and sex, as well as multivariate analysis adjusted for ADNC. Several studies demonstrated the importance of brain cholesterol homeostasis and cognition (Li et al., 2022). *ApoE* knockout mice show decreased brain cholesterol levels alongside a decline in learning and memory, suggesting that cholesterol deficiency is associated with impaired learning (Fuentes et al., 2018, Lane-Donovan et al., 2016, Nunes et al., 2018). An increase in cholesterol could be attributed to decreased cholesterol catabolism. We previously demonstrated a decrease in 24OHC levels in the CSF, suggesting a decrease in CYP46A1 activity (Simpson et al., 2016). In mice, silencing of *Cyp46A1,* leading to accumulation of cholesterol in hippocampal neurones, contributed to atrophy of the hippocampus by inducing apoptosis of hippocampal neurones (Djelti et al., 2015). Although results vary, these studies demonstrate the importance of maintaining optimum cholesterol level to prevent cognitive decline (Schultz et al., 2018).

We did not find a significant effect of *APOE* status on brain cholesterol concentration in *this* study, although *APOE* status was only available on part of the cohort. *APOE* status may have peripheral effects relevant to AD risk, as total serum cholesterol is higher in those carrying the *APOE ε4* allele (Dunk et al., 2022). In the brain, APOE is mainly expressed in astrocytes and microglia; however, neurones are also able to express this gene during AD, although at a lower level than astrocytes (Kim et al., 2009, Metzger et al., 1996, Xu et al., 2006). APOE interacts with Aβ (Karch and Goate, 2015, Kim et al., 2009) and is important for the transport of cholesterol from astrocytes to neurones (Pfrieger, 2003). Several studies have reported the importance of APOE in cholesterol metabolism and the effects of the ε4 allele on decreased cholesterol transport to neurones and cognitive decline (Ali et al., 2018, Fuentes et al., 2018, Lane-Donovan et al., 2016, Li et al., 2022, Nunes et al., 2018). Using a single cell transcriptomic approach, *APOE ε4* was shown to be associated with changes in signalling associated with cholesterol homeostasis and transport, and in altered localisation to oligodendrocytes and impaired myelination (Blanchard et al., 2022). This implies an important role in white matter, whilst our study was focused on cortical cholesterol measures. These studies show important effects of *APOE* status on cholesterol both peripherally and in brain, suggesting a need to extend cholesterol measures to brain areas other than cortex (as examined in *this* study) and for finer cell-type specific measures.

As *this* study used an ageing population-representative neuropathology cohort, it provides an unbiased assessment of cholesterol-related measures across the ADNC spectrum, which enables the investigation of cell pathology and dementia status separately without any assumptions from preselection into clinico-pathological groups (Wharton et al., 2011). *This* study also measured the changes in neuronal and astrocytic cholesterol biosynthetic gene expression showing cell-specific effects rather than overall effects, which have been the focus of many previous studies. A limiting factor in the study was sample size. For the association of brain cholesterol and cholesterol, point estimates showed increased risk of dementia in moderate and high cholesterol groups when measured in tertiles. When modelled continuously no effect was seen in cholesterol. It should be noted that the 95% CI around the estimates for the tertile analysis are wide, indicating uncertainty around the estimate and the possibility of no effect is not excluded. In contrast the estimate for continuous cholesterol measures shows no effect with a more precise 95% CI. The sample size for cholesterol measurement is small (n = 63), so that a study with a larger sample size and greater statistical power would be warranted to confirm these effects with greater certainty. There was also a small sample size for the RNA studies, which may have impaired the power to detect effects associated with dementia. Changes in relation to measures of pathology were detected, but some of the effects here were small with a lot of variation, so that a larger study would be of value to confirm and extend these relationships. Only temporal cortex was analysed from the CFAS cohort, and it is unclear whether alterations in gene expression and cholesterol with ADNC and dementia may vary between different neuroanatomical regions, and between grey and white matter regions. HMGCR protein expression was assessed using immunohistochemistry, which does not provide a linear assessment of protein concentration and cannot provide a measure of enzyme activity (Matkowskyj et al., 2003), although we looked quantitatively at mRNA to assess gene expression, which did reveal significant changes. Gene expression data was calculated relative to the expression of β-actin over Braak NFT group 1 using the 2^-ΔΔCt^ method, which is not an absolute measurement of *HMGCR* expression (Rao et al., 2013). Tissue cholesterol concentration was determined in whole temporal cortex rather than an enriched population of neurones, so that cells other than neurones contribute to the total cholesterol concentration. Furthermore, we have not examined white matter, which is an important site for the potential effects of cholesterol dysmetabolism.

## 5 Conclusions

The rate-limiting enzymes for cholesterol biosynthesis are expressed in neurones in vivo*.* ADNC is associated with gene expression changes that may impair cholesterol biosynthesis in neurones, but not astrocytes. Total cholesterol levels in temporal cortex are altered in those with a dementia phenotype but do not relate directly to pathological measures and the study did not find an effect of *APOE* status. Dysregulation of cholesterol metabolism is important in dementia but relationships to cellular pathology and functional outcome are complex and vary between cell types. It is important that future work is carried out to further understand the mechanisms of brain cholesterol homeostasis (synthesis, transport, intracellular flux, degradation) in ageing and disease and the specific dynamics on different cell types and subcellular compartments in different brain regions.

## CRediT authorship contribution statement

**Ahamed Saif U:** Investigation, Methodology. **Ashford Bridget:** Investigation, Methodology. **Matthews Fiona E.:** Data curation, Formal analysis, Writing – review & editing. **Moore Zoe:** Investigation, Methodology. **Mistry Hemant:** Investigation, Methodology, Writing – original draft. **Higginbottom Adrian:** Methodology, Supervision. **Richardson Connor D.:** Data curation, Formal analysis. **Simpson Julie E.:** Methodology, Project administration, Supervision, Writing – review & editing. **Brayne Carol:** Conceptualization, Writing – review & editing. **Wharton Stephen B.:** Conceptualization, Data curation, Formal analysis, Funding acquisition, Project administration, Supervision, Writing – original draft, Writing – review & editing.
