# Supplementary material for: Relationships of brain cholesterol and cholesterol biosynthetic enzymes to Alzheimer’s pathology and dementia in the CFAS population-derived neuropathology cohort
Source: Neurosci Res. 2024 Jul;204:22–33. doi: 10.1016/j.neures.2024.01.003 (PMC11192635; doi:10.1016/j.neures.2024.01.003)
Supplement: Supplementary file 1 — Supplementary material [file mmc1.docx]

**Supplementary Figure 1.** Cholesterol Biosynthesis Pathway.

**
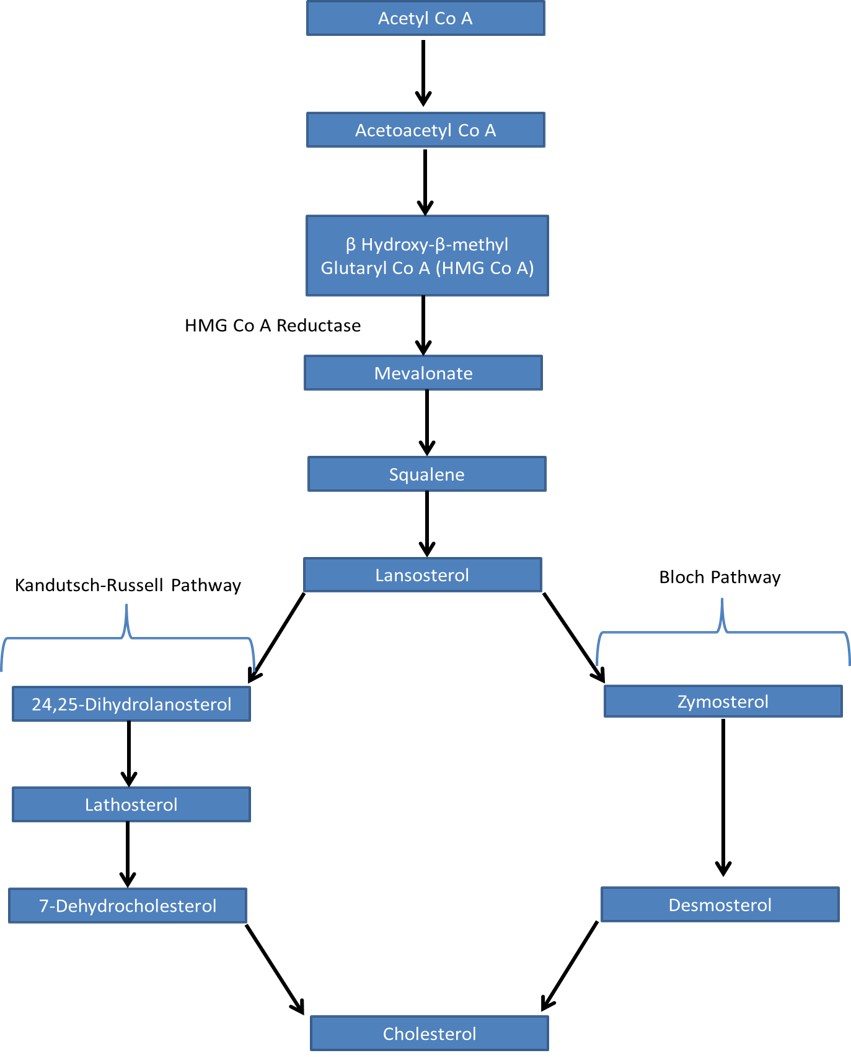
**

This diagram shows a brief summary of the cholesterol synthesis pathway. Condensation of Acetyl Co A into mevalonate is known as the mevalonate pathway. Post-squalene cholesterol production can occur via the Kandutsch-Russell or Bloch pathways.

**Supplementary Figure 2:** HMGCR expression in frozen tissue sections.

**
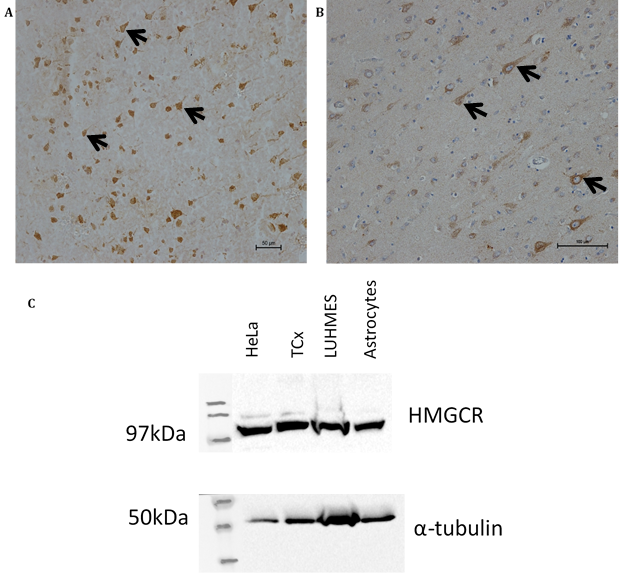
**

HMGCR expression was validated by carrying out the optimised staining protocol in frozen tissue (A). HMGCR expression was associated with pyramidal neurons of the temporal cortex in frozen tissue sections (black arrows). HMGCR immunoreactivity was punctate and found within the cytoplasm of the cell soma and proximal processes (black arrows) HMGCR immunoreactivity was detected in the cytoplasm of the neuronal cell bodies and proximal processes. Scale bar represents 50μm.

**Supplementary Figure 3**

**
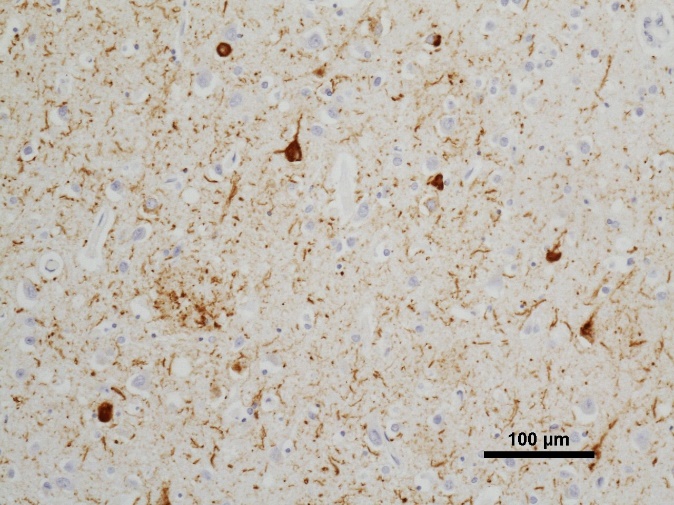

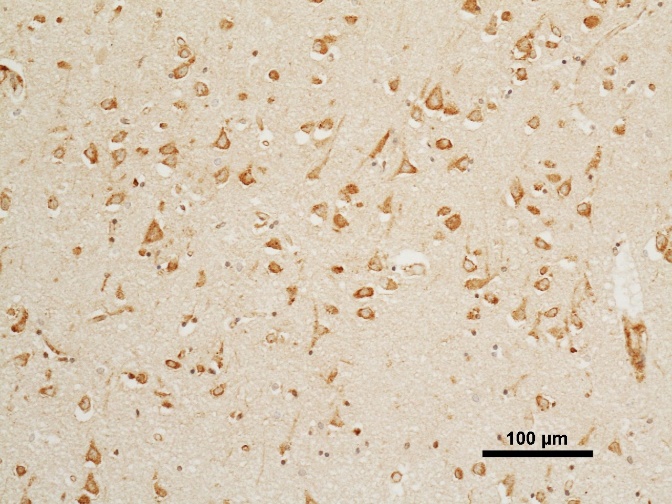
**

HMGCR expression (left) and neurofibrillary tangles identified by AT8 antibody (right) in the same cortical area. All of the neurones in the field expression HMGCR, irrespective of whether tangles are present.

**Supplementary figure 4.** Boxplots showing changes in *SREBP2* and *HMGCR* mRNA levels, corrected for pyramidal neuronal counts, with local temporal cortex CERAD-type ADNC measures for tangles and neuritic plaques.


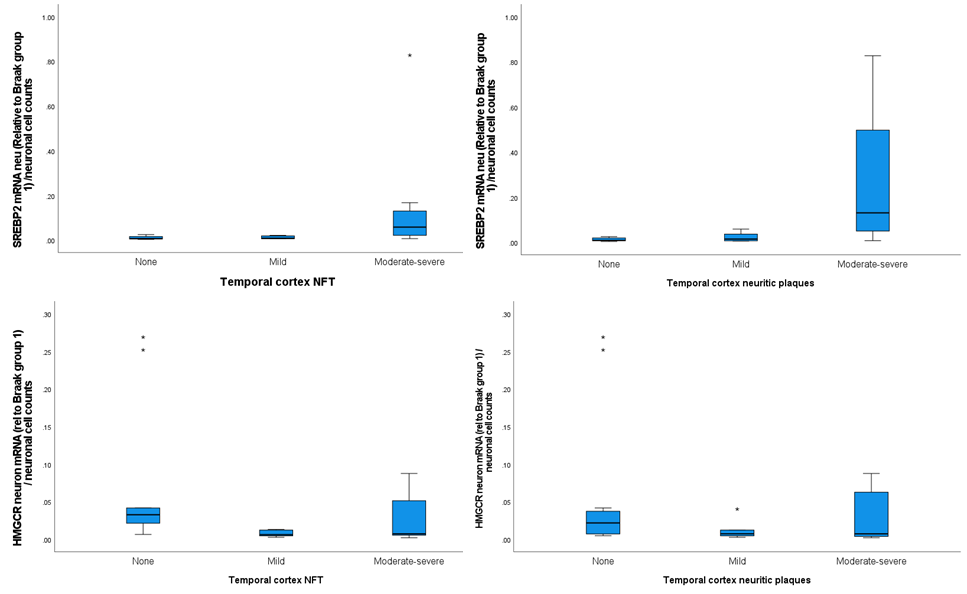


**Supplementary Table 1.** Characteristics of sample for cholesterol and relation to *APOE* genotype

| **Cholesterol and *APOE* sample** | **n** | **Proportion (%)** | **Mean cholesterol** | **Standard error** | ***p** |
| --- | --- | --- | --- | --- | --- |
| **Cholesterol concentration µM/mg tissue** | 61 |  | 14.58 | 7.14 |  |
| **Cholesterol tertiles** |  |  |  |  |  |
| Low | 22 | 36.07 | 7.18 | 0.65 |  |
| Moderate | 19 | 31.15 | 14.44 | 0.47 |  |
| High | 20 | 32.79 | 22.85 | 0.75 |  |
| ***APOE* genotype** |  |  |  |  |  |
| 2.2 | 0 | - | - | - |  |
| 2.3 | 6 | 10.53 | 13.12 | 3.83 |  |
| 2.4 | 3 | 5.26 | 17.37 | 5.93 | 0.67 |
| 3.3 | 27 | 47.37 | 14.40 | 1.48 |  |
| 3.4 | 20 | 35.09 | 14.77 | 1.43 |  |
| 4.4 | 1 | 1.75 | 6.59 | - |  |
| ***APOE4 Negative*** | 33 | 57.89 | 14.16 | 1.37 | 0.76 |
| ***APOE4 Positive*** | 24 | 42.11 | 14.75 | 1.33 |  |

*Kruskal Wallis

**Supplementary Table 2. Tissue cholesterol concentration does not vary with ADNC or other cellular pathologies**

| **Pathology** | **Measures** | | | | | | | | | | | | | | |
| --- | --- | --- | --- | --- | --- | --- | --- | --- | --- | --- | --- | --- | --- | --- | --- |
| **ADNC** | Braak stage  KW p=0.401  JT p=0.543 | | | Thal phase  KW p=0.634  JT p=0.985 | | | | Aβ IR  r=0.095  p=0.478 | | | | | AT8 (tau) IR  R=0.095  P=0.476 | | |
| **Oxidative stress** | γH2Ax Neur  r=0.117  p=0.374 | | | | | γH2Ax Ast  r=0.047  p=0.723 | | | | | Malondialdehyde  r=0.245  p=0.298 | | | | |
| **Neuroinflammation** | CD68  r=-0.011  p=0.940 | | | | | MHCII  r=0.125  p=0.377 | | | | | GFAP  r=0.043  p=0.745 | | | | |
| **Vascular** | Cerebral amyloid angiopathy  KW p=0.705  JT p=0.603 | | | | | | | Cortical microinfarcts  MW p=0.735 | | | | | | | |
| **Cholesterol homeostasis** | HMGCR IR r=0.237  P=0.061 | 24-OHC CSF  R=0.34  P=0.442 | mRNA | | | | | | | | | | | | |
|  |  |  | HMGCR  Ast  r=-0.286  p=0.535 | | HMGCR  Neur  r=0.217  p=0.576 | | SREBP2  Ast  r=0.600  p=0.285 | | SREBP2 Neur  r=0.517  p=0.154 | ABCA1 Ast  r=0.600  p=0.400 | | ABCA1 Neur  r=0.300  p=0.624 | | CYP46A1 Ast  r=0.800  p=0.200 | CYP46A1 Neur  r=0.146  p=0.669 |

A summary of the various measures assessed to determine relationships of tissue cholesterol concentration with various markers of ADNC, neuroinflammation and oxidative stress. Where r is the Spearmen’s rank correlation coefficient and p is the significance value. The table also shows the p values for KW (Kruskal-Wallis), JT (Jonckheere-Terpstra) and MW (Mann-Whitney U) tests. All tests were two tailed and the p<0.05 was set as the level for significance. IR - % area immunoreactivity. 24-OHC CSF – 24 hydroxycholesterol assessed in cerebrospinal fluid.
